# Fascial Pathophysiology in Hypermobility Spectrum Disorders and Hypermobile Ehlers–Danlos Syndrome: A Review of Emerging Evidence

**DOI:** 10.3390/ijms26125587

**Published:** 2025-06-11

**Authors:** Tina J. Wang, Antonio Stecco, Alan J. Hakim, Robert Schleip

**Affiliations:** 1 Department of Physical Medicine and Rehabilitation, Loma Linda University School of Medicine, Loma Linda, CA 91786, USA; 2 Department of Physical Medicine and Rehabilitation, New York University Grossman School of Medicine, New York, NY 10016, USA; antonio.stecco@nyulangone.org; 3 Department of Neurology, University College London Hospitals, London WC1N 3BG, UK; alan.hakim@nhs.net; 4 Department of Applied Physiology, University of Ulm, 89081 Ulm, Germany; robert.schleip@uni-ulm.de

**Keywords:** fascia, hypermobility, Ehlers–Danlos syndromes, HSD, extracellular matrix, mechanobiology, connective tissue disorders, neuroimmune inflammation, proprioception, autonomic dysfunction

## Abstract

Hypermobile Ehlers–Danlos syndrome (hEDS) and hypermobility spectrum disorders (HSD) are increasingly recognized as complex, multisystem connective tissue disorders characterized by joint hypermobility and instability, chronic pain, autonomic dysfunction, immune dysregulation, and structural fragility. Despite their clinical impact and prevalence, the underlying pathophysiology remains poorly understood, and diagnosis is frequently delayed or missed altogether. Emerging research highlights the fascia as a central player in the pathogenesis of these conditions. This narrative review synthesizes current molecular, histological, and biomechanical findings to propose a fascia-centered framework for understanding hEDS and HSD. Evidence from transcriptomic and imaging studies reveals consistent abnormalities in fascial thickness, interfascial gliding, myofibroblast activation, tendon elongation, and tissue stiffness—findings that mirror the functional impairments reported in clinical populations. We explore fascia as a dynamic tissue network and consider how dysregulation in these processes may contribute to the widespread symptoms seen in hypermobility disorders. By reframing hEDS and HSD as disorders of pathological fascial remodeling, this review offers an integrated model that connects molecular mechanisms with clinical expression. It underscores the urgent need for multidisciplinary research to define diagnostic biomarkers, clarify therapeutic targets, and support the development of more effective, personalized interventions.

## 1. Introduction

Ehlers–Danlos syndromes (EDS) and hypermobility spectrum disorders (HSD) are heritable connective tissue disorders characterized by joint hypermobility and instability, skin hyperextensibility, and connective tissue fragility [1]. The clinical burden is often significant and debilitating, with patients experiencing chronic musculoskeletal pain, joint instability, proprioceptive deficits, and frequent injuries [2]. While these conditions are traditionally classified as musculoskeletal connective tissue disorders, EDS and HSD are now increasingly recognized as multisystemic conditions. The wide spectrum of systemic manifestations include autonomic dysregulation, immune and gastrointestinal dysfunction, chronic fatigue, and widespread pain [2,3]. The involvement of these extra-musculoskeletal systems further complicates the clinical picture, often resulting in delayed or missed diagnoses and profound reduction in quality of life [4].

The multisystemic nature of EDS and HSD underscores the need to move beyond a compartmentalized, tissue-specific model of pathophysiology. Instead, it supports an emerging understanding that fascial system pathology may underlie or contribute to the diverse presentations observed in these conditions. Given fascia’s integral role in structural support, mechanotransduction, proprioception, and systemic communication, fascial dysfunction offers a compelling unifying framework for understanding the widespread manifestations of EDS and HSD.

### 1.1. Nosology of Hypermobility Disorders

Hypermobility syndromes represent a broad and evolving nosological landscape, increasingly understood through the lens of fascial system pathology. Historically considered a subset of connective tissue disorders, these conditions are now being reconceptualized to reflect the integral role of the extracellular matrix (ECM) of the fascial system in their pathogenesis. As the significance of fascial pathology becomes more widely recognized, the taxonomy and classification of hypermobility disorders are expanding to include a wider spectrum of related conditions. For example, periodontal Ehlers–Danlos syndrome—a subtype characterized by complement dysfunction—demonstrates features of connective tissue fragility and ECM disruption, further blurring the lines within the broader EDS and HSD spectrum with autoimmune conditions [5].

The presentation of joint hypermobility ranges from non-clinical asymptomatic generalized joint hypermobility to more complex clinical and multisystemic disorders encompassed by HSD and EDS. Among the 13 recognized types of EDS, hypermobile EDS (hEDS) is the most prevalent. However, it remains the most enigmatic due to the absence of an identifiable genetic marker. Diagnosis of hEDS is currently based on clinical criteria, including a positive Beighton score, chronic musculoskeletal pain or joint instability, and evidence of connective tissue fragility [1].

Distinguishing hEDS from HSD remains particularly challenging due to significant phenotypic overlap and the lack of definitive molecular diagnostics [6]. This diagnostic ambiguity leaves many patients in a gray zone, often cycling through years of misdiagnosis or underdiagnosis. Emerging evidence suggests that hEDS and HSD may lie on a phenotypic continuum of a shared underlying pathology, rather than being entirely distinct entities. Central to this evolving model is the dysregulation of the ECM and aberrant fibroblast-to-myofibroblast transition—a cellular mechanism typically activated during tissue repair, wound healing, and fibrosis [7].

In individuals with hEDS and HSD, this transition appears to be pathologically sustained, leading to chronic, low-grade inflammation, tissue laxity, and persistent nociceptive and neuropathic pain. The resulting state of fascial and ECM dysfunction results in system-wide manifestations and the high burden of morbidity observed in affected individuals. These insights also offer a foundation for future research into targeted therapies, diagnostics, and a more nuanced nosological framework for hypermobility disorders [7].

### 1.2. Rationale for a Fascia-Centered, Molecular-Level Review

Given the systemic and heterogeneous nature of hypermobility-related disorders, a growing body of evidence points to the fascial system as a critical, yet underrecognized, site of pathophysiological change. Fascia is not merely a structural support matrix—it is a highly dynamic, innervated, and vascularized connective tissue network involved in force transmission, proprioception, pain modulation, and intercellular communication. Its responsiveness to mechanical and biochemical stimuli implicates it in a range of processes central to health and disease, including inflammation, fibrosis, and neurosensory dysregulation [8].

The multilayered nature of fascia—including superficial, deep, visceral, and neural components—offers a plausible anatomical basis for the systemic manifestations observed in disorders such as hEDS and HSD [9]. Dysfunctions in this network, particularly those involving fibroblast-to-myofibroblast transition, ECM remodeling, and aberrant mechanotransduction, may drive many of the clinical features in hypermobility disorders.

Moreover, parallels are emerging between hEDS/HSD and other underexplored connective tissue disorders—such as Dercum disease and lipedema—which also exhibit features of fascial involvement, tissue fragility, and pain, despite lacking formal recognition within existing nosologies [10,11,12]. These overlaps underscore the need for a unifying framework that moves beyond symptom-based classification and toward a shared molecular and fascial pathophysiology.

This review therefore proposes a fascia-centered, molecular-to-anatomic level approach to understanding hypermobility syndromes and related connective tissue disorders (Table 1). By examining current evidence on fibroblast dysfunction, ECM dysregulation, and altered fascial architecture, we aim to provide a cohesive fascial based model that better explains the clinical complexity and guides future research and therapeutic strategies.

### 1.3. Aim and Scope of the Review

This review aims to synthesize current scientific understanding of hypermobility disorders through the evolving framework of fascial biology and science, with an emphasis on shared molecular mechanisms and pathophysiological pathways. Specifically, the objectives of this review are to:

1. Provide an overview of the clinical and molecular features of EDS and HSD, with particular focus on hEDS.
2. Examine the role of fascia—particularly fibroblast dysregulation and ECM remodeling—in the pathogenesis of hypermobility syndromes.
3. Explore converging molecular mechanisms, including abnormal myofibroblast persistence, matrix degradation, and low-grade inflammation, as drivers of systemic dysfunction.
4. Highlight anatomical and clinical consequences of these molecular changes, and explore shared fascial features with related connective tissue disorders such as Dercum disease and lipedema.
5. Identify current gaps in the literature and propose directions for future research, diagnostic refinement, and targeted therapeutic strategies.

## 2. Clinical and Molecular Landscape of HSD/hEDS

### 2.1. Diagnostic Criteria

HSD and hEDS represent the most prevalent presentations within the broader category of heritable connective tissue disorders. Characterized by joint hypermobility, joint instability, soft tissue fragility, and chronic musculoskeletal pain, these conditions also manifest with a wide array of systemic features. Despite their substantial disease burden, both hEDS and HSD remain difficult to diagnose due to the absence of genetic or other molecular markers, variability in clinical presentation, and evolving classification systems.

Among the 13 recognized EDS types, hEDS is unique in that it lacks a known genetic etiology, making it the only type diagnosed solely on clinical presentation. In contrast, all other EDS types are linked to variants in genes associated with collagen biosynthesis or other components of the ECM. This lack of a known genetic etiology places considerable emphasis on physical examination, symptom reporting, and the exclusion of alternative diagnoses. In practice, the absence of objective biomarkers complicates the clinical picture and contributes to both underdiagnosis and misdiagnosis [13].

To address the need for diagnostic clarity, the International Consortium on the Ehlers–Danlos Syndromes and Hypermobility Spectrum Disorders introduced a revised classification system in 2017. This framework was developed to differentiate hEDS from other connective tissue disorders, including HSD and non-syndromic joint hypermobility, and to standardize clinical assessments. According to these guidelines, the diagnosis of hEDS requires fulfillment of three core criteria. First, generalized joint hypermobility must be confirmed, typically using the Beighton scoring system, with adjusted thresholds based on age. Second, patients must exhibit at least two of the following: systemic features suggestive of connective tissue disorder, such as skin hyperextensibility or atrophic scarring; a positive family history in a first-degree relative; or a history of musculoskeletal complications such as joint instability or recurrent dislocations without trauma. Third, other heritable and acquired connective tissue disorders must be ruled out as the cause for symptom presentation, including genetically defined forms of EDS, Marfan syndrome, Loeys-Dietz syndrome, and autoimmune or inflammatory disorders that may have hypermobility-related symptoms and traits as part of the disease process [1].

In contrast to hEDS, the diagnosis of HSD is reserved for individuals who exhibit symptomatic joint hypermobility but do not meet the full diagnostic criteria for hEDS. HSD is a broader, more inclusive classification intended to capture individuals whose symptoms are clinically significant but fall outside the formal hEDS framework. The diagnostic criteria for HSD emphasize functional impairment, with joint instability, dislocations, or chronic pain that interfere with daily life. HSD may present as generalized, peripheral, localized, or historical, depending on the pattern and distribution of symptoms. While the 2017 classification system clarified distinctions between hEDS and HSD on paper, the reality is that the two conditions often exist on a clinical continuum, with considerable overlap in presentation and disease burden [14].

Specifically, autoimmune conditions have been observed at a disproportionately higher rate among individuals with HSD and hEDS [15]. While the presence of a concurrent autoimmune connective tissue disorder such as rheumatoid arthritis or systemic lupus erythematosus, for example, does not exclude an HSD or hEDS diagnosis, more stringent criteria are required when considering a diagnosis of hEDS in such cases. Specifically, a documented family history of documented hEDS diagnosis becomes essential to support a diagnosis of hEDS in the presence of an autoimmune connective tissue condition. This requirement reflects the considerable clinical overlap between hEDS and such autoimmune disorders, both of which are frequently associated with joint pain and systemic symptoms, making differential diagnosis particularly challenging [16,17]. Therefore, when an autoimmune connective tissue condition is present, a diagnosis of hEDS should be made cautiously and only when the clinical presentation aligns with established diagnostic criteria and a supporting family history.

Ultimately, the current diagnostic criteria—while an important step toward standardization—still rely heavily on subjective findings and symptom exclusion. Without a genetic or molecular marker, clinical diagnosis remains challenging, particularly in patients with subtle or evolving symptoms. The substantial phenotypic overlap between hEDS, HSD, autoimmune connective tissue disorders, and other heritable connective tissue disorders, including those not formally classified within the EDS spectrum, highlights the need for a more mechanistically informed diagnostic framework.

### 2.2. Pathological Myofibroblast Transition in hEDS and HSD

In hEDS and HSD, fibroblast-to-myofibroblast transformation becomes dysregulated. Transcriptomic studies reveal a distinct cellular phenotype compared to other EDS types, with extensive dysregulation of genes involved in ECM remodeling, cytoskeletal integrity, integrin signaling, and immune modulation [7,18,19].

Fibroblasts are mesenchymal-derived tissue-resident cells that play a primary role in ECM synthesis, maintenance, and mechanical sensing [20,21]. They are present in all organ systems and are particularly responsive to biomechanical cues such as stretch or shear, which can induce cytoskeletal remodeling and undergo phenotypic changes to myofibroblasts [22,23]. In addition to their structural role, fibroblasts also function as immune sentinel cells, activated by both innate and adaptive immune pathways. Upon injury, they secrete cytokines that recruit and regulate immune cells such as macrophages, thereby influencing local inflammation and wound repair [24,25,26].

While initial studies identified dysregulated fibroblast-to-myofibroblast transitions in dermal tissue, other investigations have demonstrated a statistically significant presence of myofibroblasts within the deep fascia of individuals with HSD and hEDS compared to those with generalized joint hypermobility and non-hypermobile controls [27]. Earlier molecular research has largely focused on skin biopsies, revealing aberrant myofibroblast differentiation [7]. However, these findings may be confounded by the high prevalence of allergic skin conditions in this population, which can produce non-specific inflammatory responses [28,29]. By focusing on deep fascial tissue, particularly in structurally load-bearing areas such as the iliotibial band, other studies eliminate these dermatological confounders and provide stronger evidence for ECM disorganization as a feature of hypermobility syndromes, supporting the hypothesis that fascial rather than purely dermal pathology plays a central role in the underlying disease mechanisms of hEDS and HSD.

One key molecular driver of myofibroblast transition and disruptions in focal adhesion structures involves the αvβ3 integrin–ILK signaling axis. The αvβ3 integrin is a cell surface receptor involved in mechanotransduction and ECM adhesion. This integrin is pathologically upregulated in hEDS/HSD fibroblasts and distributed in fibrillar and focal adhesion complexes across the cell surface [7,19].

Upon activation, αvβ3 integrins initiate intracellular signaling cascades through integrin-linked kinase and downstream transcription factors Snail1 and Slug, ultimately promoting the expression of α-smooth muscle actin (α-SMA) and converting fibroblasts into myofibroblasts [18,19]. In hEDS/HSD, between 60 and 90% of fibroblasts express α-SMA, a stark contrast to controls, indicating a widespread shift toward a pro-fibrotic phenotype. These myofibroblasts exhibit resistance to apoptosis (anti-anoikis), allowing them to persist in tissue longer than normal, continuously contributing to ECM stiffness and dysfunction. These altered adhesion complexes enhance migratory capacity by fivefold and facilitate the spread of disordered matrix deposition. Integrin receptors such as α2β1 and α5β1, essential for matrix anchoring, also show reduced expression, further weakening ECM integrity and altering cellular feedback loops [7,19].

Integrin activation can also influence the Wnt/β-catenin signaling pathway—a master regulator of ECM gene expression. This pathway, once activated, enhances protease activity, disrupts ECM architecture, and contributes to increased elasticity and softening of the connective tissue, consistent with the tissue phenotype seen in hEDS/HSD [30].

Adding another layer of complexity, recent studies suggest that epigenetic mechanisms—particularly through microRNAs (miRNAs)—may regulate fibroblast behavior in hEDS/HSD. The dysregulation of miRNA expression—such as the overexpression of miR-378-3p and miR-224 and downregulation of miR-23a—has been observed. The latter is particularly important, as miR-23a downregulation is associated with upregulation of Wnt pathway activation, providing a potential epigenetic link to sustained myofibroblast activity [18].

### 2.3. ECM Remodeling and Dysregulation in hEDS and HSD

Central histological findings of hEDS and HSD are widespread ECM disorganization, chronic inflammation, and tissue fragility. The ECM is a highly organized, three-dimensional macromolecular network that provides mechanical support to tissues and serves as a biochemical signaling reservoir. It consists primarily of collagens, glycoproteins (including fibronectin, laminins, and tenascin), proteoglycans, elastin, and glycosaminoglycans such as hyaluronic acid—the most abundant glycosaminoglycan in the matrix. The ECM also harbors bioactive molecules including cytokines, growth factors (e.g., TGF-β1), and matricellular proteins like osteopontin and tenascins, which regulate cellular function, migration, differentiation, and immune responses [31,32].

Continuous ECM remodeling is essential for normal tissue maintenance and repair. Dysregulation of this process, however, is closely associated with fibrotic and inflammatory diseases [33,34]. In hEDS and HSD, growing evidence indicates that ECM homeostasis is severely disrupted, creating a pathological tissue microenvironment that drives persistent fibroblast activation and transformation.

ECM from hEDS and HSD patients show disarray of collagen types I, III, and V—key components that provide tensile strength and integrity to connective tissues. Elastin, another essential ECM protein that imparts elasticity, is aberrantly localized within the cytoplasm of fibroblasts rather than being secreted into the ECM, suggesting impaired elastogenesis [18,35].

Proper fibril connections to the ECM are notably absent in hEDS and HSD fibroblasts, compromising the biomechanical stability of the matrix. Fibrillins, which are required for the formation of elastic fibers and microfibrillar scaffolds, are often undetectable. Additionally, fibronectin and tenascin—critical for ECM organization, tissue repair, and cell adhesion—fail to integrate into the ECM, indicating a disruption in matrix maturation and stability [18,35].

There is a marked decrease in the expression of receptors essential for ECM-cell communication, including collagen type I and fibrillin receptors, as well as integrins α2β1 and α5β1. These receptors play pivotal roles in cellular adhesion, migration, and mechanotransduction. Their downregulation contributes to the defective cell–ECM interactions observed in hEDS and HSD, further impairing matrix remodeling and tissue repair processes [18,35].

Within the disorganized ECM characteristic of hEDS and HSD, a unique biochemical signature has been identified: a 52 kDa fibronectin breakdown product. This fragment appears to be specific to individuals with HSD and hEDS, and has not been observed in other EDS types or in healthy controls [36].

Fibronectin is a large, multifunctional glycoprotein essential for cell adhesion, migration, differentiation, and matrix organization. It exists in both soluble (plasma) and insoluble (ECM-integrated) forms and is a key component of the scaffold that organizes collagens, elastin, and integrins into a cohesive structural network. It also interacts with growth factors and cell surface receptors, playing a pivotal role in mechanotransduction and tissue homeostasis [37,38].

Under physiological conditions, fibronectin is synthesized by fibroblasts and assembled into fibrillar structures that support mechanical integrity and intercellular signaling. However, in hEDS and HSD, fibronectin is frequently misassembled, fragmented, or absent from the ECM entirely. Transcriptomic and proteomic studies have shown a failure of fibronectin to properly incorporate into matrix fibers, contributing to a structurally weakened and functionally disrupted ECM [18,35].

Together, these abnormalities underscore a fundamental disruption in ECM architecture and signaling in hEDS and HSD, providing a histological basis for the widespread connective tissue fragility and impaired healing observed in these disorders.

### 2.4. Immune–Fibroblast Crosstalk and Immune Dysregulation in hEDS and HSD

One of the more commonly occurring clinical features of hEDS and HSD is a chronic state of immune dysregulation, marked by a paradoxical combination of immune deficiency and allergic or inflammatory hypersensitivity [39]. Though seemingly disparate, these abnormalities converge to promote persistent inflammation and aberrant tissue remodeling. This dysregulated immune environment is further sustained by intense crosstalk between immune cells and fibroblasts, forming a self-perpetuating loop. The resulting interaction not only maintains the fibroblast-to-myofibroblast transition but also amplifies ECM degradation and immune activation, contributing to the multisystemic and progressive nature of both conditions.

Mast cells are increasingly recognized as critical mediators of fibroblast behavior in connective tissue disorders. These immune cells are abundant in the dermis, gastrointestinal tract, and fascial tissues—the same structures often affected in hEDS and HSD [29]. They become activated via IgE receptor cross-linking, leading to degranulation and the release of pro-inflammatory mediators, including histamine, tryptase, cytokines, and growth factors [6,40].

At the center of this feedback loop is the crosstalk between immune cells and myofibroblasts, which drives continued fibrosis, pain, and systemic symptoms. Macrophages release TGF-β1 and other cytokines in response to tissue injury and stress, promoting myofibroblast differentiation and excessive ECM production [41]. Mast cells, frequently activated in hEDS and HSD, release histamine, tryptase, and pro-inflammatory cytokines that amplify the inflammatory response, recruit additional immune cells, and increase vascular permeability and tissue edema [42]. Myofibroblasts themselves produce cytokines such as IL-6 and monocyte chemoattractant protein-1 (MCP-1), which promote T-cell activation and further immune cell recruitment [43,44].

One important pathway linking mast cells to ECM remodeling is through αvβ3 integrins. Abnormally elevated αvβ3 integrin expression, as observed in hEDS/HSD fibroblasts, may not only promote myofibroblast differentiation but also alter mast cell behavior, facilitating a bidirectional loop of activation. Upon degranulation, mast cell-derived mediators further destabilize integrins, disrupt ECM integrity, and promote additional fibroblast-to-myofibroblast transitions, thereby sustaining a pathological remodeling environment [7,45,46].

As the ECM becomes disorganized, it begins to release matrix-derived danger-associated molecular patterns (DAMPs), including fragmented fibronectin, collagen type I, and tenascin, as well as matrix metalloproteinase-9 (MMP) and matricellular proteins like CCN1/CYR61 and CCN2/CTGF. These ECM fragments act not only as structural debris but also as bioactive molecules that amplify inflammation and signal further myofibroblast conversion. DAMPs engage toll-like receptors on immune cells, particularly macrophages and dendritic cells, initiating pro-inflammatory cascades that further perpetuate tissue breakdown [7,40,45,46].

Transcriptomic analyses in hEDS and HSD reveal significant dysregulation in immune-related gene expression. This includes the upregulation of TGF-β, a potent profibrotic cytokine, as well other matricellular proteins such as histones, spondin-2 (SPON2), and filaggrin (FLG)—each implicated in autoimmune or allergic responses [18,35,39,47]. Notably, histones, normally sequestered within the nucleus, can act as potent DAMPs when extracellular, and have been implicated in systemic autoimmune diseases such as rheumatoid arthritis and systemic lupus erythematosus [47].

SPON2, an ECM-associated protein upregulated in hEDS/HSD, is known to regulate innate immune responses through its interaction with macrophages and stimulation of pro-inflammatory cytokine release. It has also been linked to allergic airway inflammation [48]. Likewise, FLG, a protein associated with skin barrier function, is overexpressed in these conditions and has been implicated in asthma, food allergies, and atopic dermatitis [49].

Conversely, some immune pathways appear to be downregulated. For instance, IL-6, a cytokine critical for immunoglobulin class switching and B-cell activation, is suppressed in hEDS/HSD [35]. The downregulation of IL-6 may be associated with the observed trend of IgG subclass deficiencies in affected individuals, contributing to frequent infections and chronic fatigue [39,50,51].

miRNAs also play a role in immune regulation in hEDS and HSD. The overexpression of miR-378-3p and miR-224 and downregulation of miR-23a are associated with the upregulation of Wnt/β-catenin signaling, indicating an epigenetic basis for the dysregulated immune–fibroblast interaction [18]. Furthermore, the downregulation of NR4A nuclear receptors, which normally act to suppress NF-κB-mediated inflammation, has been reported in hEDS/HSD. Loss of this inhibitory control may contribute to the sustained activation of pro-inflammatory pathways [52].

Additional evidence for immune system involvement comes from the upregulation of complement cascade components in hEDS/HSD tissues. These include complement factor D, complement C1r, C9, C4b-binding protein alpha chain, and vitronectin—proteins central to both innate immunity and immune complex clearance [53]. Dysregulation of the complement system is associated with various autoimmune and inflammatory diseases, including rheumatic disease and sepsis [54] as well as periodontal EDS [5].

The prolactin receptor (PRLR) is also upregulated in hEDS/HSD. Prolactin, traditionally known as a hormone, also functions as a cytokine-like immune modulator. PRLR is expressed on a variety of immune cells, and prolactin itself can be produced by these cells in an autocrine/paracrine fashion. Elevated prolactin levels have been linked to autoimmune conditions such as systemic lupus erythematosus and rheumatoid arthritis [55].

These findings reveal a complex and reciprocal relationship between the immune system and fibroblast activity in hEDS and HSD. The interplay between mast cells, matrix fragments, integrins, and immune signaling molecules sustains a chronic inflammatory microenvironment and fuels the progression of ECM degradation and tissue fragility. Transcriptomic and proteomic data further support a model in which innate and adaptive immune dysfunction, epigenetic alterations, and matrix-derived signals converge to drive a self-perpetuating pathological loop. These insights not only help explain the multisystem clinical features of hEDS and HSD but also identify potential molecular targets for future therapeutic intervention.

### 2.5. Molecular Triggers of Myofibroblast Differentiation: The Role of TGF-β, Autonomic Dysfunction, and Inflammation

In hEDS and HSD, the pathological transformation of fibroblasts into myofibroblasts is a central mechanism driving ECM remodeling, tissue stiffening, and chronic pain. Among the molecular triggers of this process, TGF-β1 plays a pivotal role, linking mechanical strain, psychological stress, and neuroimmune signaling into a self-reinforcing loop of dysfunction.

Approximately 60% of individuals with HSD or hEDS experience dysautonomia, often presenting as orthostatic intolerance, fatigue, and temperature dysregulation [56]. This autonomic involvement is likely rooted in the unique anatomical and functional features of the fascial system, which is richly innervated by autonomic nerve fibers. Autonomic nerves account for approximately 33.8% to 40% of all nerve fibers within fascia, with the highest densities found in the surrounding blood vessels, where they are involved in vasomotor regulation and vascular constriction [57,58]. There is also a direct sympathetic innervation of intrafusal muscle fibers [59].

The impact of psychological stress on connective tissue biology is particularly relevant in individuals with hEDS and HSD, who frequently experience high levels of anxiety, depression, and somatization [60,61]. Chronic emotional stress is not an isolated experience—it has biochemical consequences that directly affect the fascial system [62,63]. Disruption of this neuromyofascial network—whether due to mechanical strain, chronic inflammation, or stress—can trigger sympathetic nervous system activation, leading to the release of TGF-β1 into the fascial microenvironment [64,65]. TGF-β1 is a potent cytokine that not only stimulates myofibroblast differentiation but also activates immune cells, thus acting as a molecular bridge between neural signaling, immune activation, and connective tissue remodeling [62,63].

**Figure 1:**
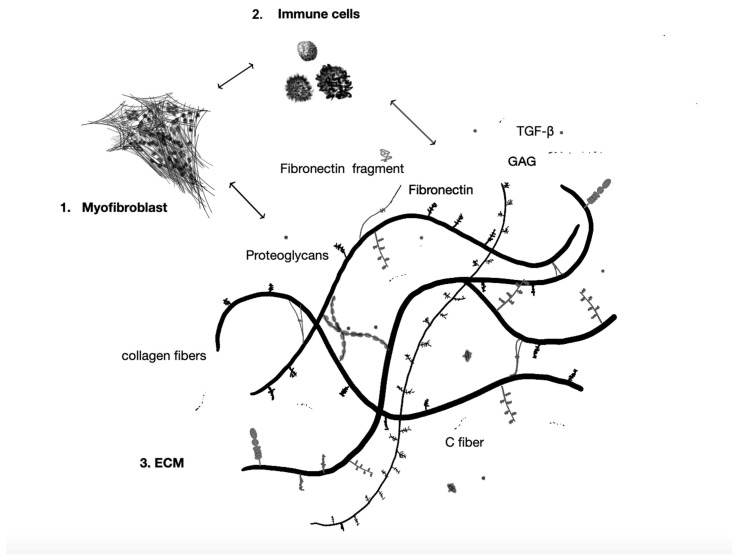
Pathways driving fascia pathology. Myofibroblast persistence, immune amplification, and ECM/neural destabilization create a self-reinforcing triad that underlies chronic fascial dysfunction in hypermobility disorders. 1. Myofibroblast Persistence. Pathological signaling through the αvβ3-integrin → ILK → Snail1/Slug axis pushes fibroblasts into a chronic myofibroblast state. These cells over-express α-SMA, evade apoptosis, migrate aggressively, and continually remodel their micro-environment. 2. Immune Amplification. Constant crosstalk with mast cells, macrophages, and T-cells floods the matrix with IL-6, TGF-β1, MCP-1, and ECM-derived DAMPs. This feed-forward loop heightens inflammation and hypersensitivity and drives yet more fibroblast-to-myofibroblast conversion. 3. ECM Breakdown and Neuro-irritation. The dysregulated myofibroblasts disrupt collagen I/III/V organization and misplace elastin and fragment fibronectin while down-regulating α2β1/α5β1 integrins, severing normal cell–matrix feedback. The resulting fragile, disorganized ECM irritates autonomic nerves; combined with TGF-β1 surges from mechanical or physiological or psychological stress, this provokes autonomic symptoms and perpetuates the cycle.

Once initiated, the mechanical and psychological triggers of fascial dysfunction activate a chronic inflammatory loop that sustains and amplifies ECM remodeling through the crosstalk between immune cells and fibroblasts. This convergence of mechanical loading, emotional stress, and immune dysregulation, all mediated through TGF-β1 signaling and myofibroblast activation, provides a unifying framework for understanding the multisystemic manifestations of hEDS and HSD (Figure 1). The resulting fascial changes, vascular dysregulation, and immune activation contribute not only to local tissue dysfunction but also to systemic symptoms such as dysautonomia, fatigue, chronic pain, and psychological distress.

TGF-β1 plays a critical role in nociceptive processing and the modulation of pain signaling. It has been shown to increase the excitability of primary sensory neurons and amplify pain responses across various pathological states. Specifically, TGF-β1 has been implicated in the regulation of cyclin-dependent kinase 5 activity, a key mediator of inflammatory pain pathways in sensory neurons [66]. Moreover, elevated levels of TGF-β1 have been associated with neuronal hyperexcitability, contributing to the development of chronic pain and hyperalgesia [67].

Chronic fatigue is a highly prevalent and debilitating symptom among individuals with hEDS, significantly impacting quality of life and functional capacity [68]. Accumulating evidence suggests that fatigue in hEDS is multifactorial in origin, involving a complex interplay between autonomic dysfunction, sleep disturbances, pain, and psychological comorbidities [69]. Fascial dysfunction and associated autonomic dysfunction is a potential etiology. Several studies have demonstrated elevated levels of TGF-β1 in patients with chronic fatigue syndrome compared to healthy controls [70]. Furthermore, there is a positive correlation between cytokine levels and disease severity [71].

By acknowledging the role of TGF-β1 as a central molecular trigger that integrates physical, emotional, and immunological stressors, this model underscores the importance of a biopsychosocial fascial based approach to diagnosis and treatment. Targeting the inflammatory and neuroimmune cascades, alongside mechanical interventions, may be critical to interrupting the pathological feedback loops that define these connective tissue disorders.

## 3. Fascia Dysfunction: From Superficial Layers to Tendons

The molecular and cellular abnormalities observed in the fascial system of individuals with EDS and HSD—including dysregulated fibroblast activity, pathological myofibroblast differentiation, and disorganized ECM remodeling—translate into visible, macroscopic alterations in fascial structure and function. These systemic tissue-level changes extend across the entire fascial continuum, from the superficial dermal fascia to the deep aponeurotic layers and even visceral fascia leading to internal dysfunction—like organ prolapses and gastrointestinal dysmotility—typical of hEDS/HSD [72].

Advances in high-resolution imaging, particularly ultrasound elastography, have enabled the visualization of these abnormalities in vivo. Findings include increased fascial thickness, reduced glide between fascial planes, and altered tissue stiffness. Such structural disruptions interfere with the fascia’s ability to coordinate movement, distribute load, and support proprioceptive function, contributing to the hallmark symptoms of joint instability, musculoskeletal pain, and impaired motor control in EDS/HSD patients. Understanding how these microscopic disruptions manifest at the tissue and organ levels is critical for linking the molecular pathology of connective tissue disorders to their clinical presentations.

### 3.1. Structural Changes in Deep Fascia in hEDS and HSD

In individuals with hEDS and HSD, the ECM undergoes pathological remodeling, resulting in profound changes to the structure and function of deep fascia. One of the features of this remodeling is an altered ECM deposition, which leads to fascial densification. Multiple ultrasound studies have demonstrated fascial alterations and dysfunction of the deep fascia in hEDS and HSD patients, particularly in regions such as the sternocleidomastoid, iliotibial tract, thoracolumbar fascia, and fascia iliaca. These findings indicate a generalized densification process in hypermobile populations [73,74,75].

This fascial densification is likely associated with a pathological fibroblast-to-myofibroblast transition and elevated inflammatory mediators, such as TGF-β1 and MMP-9. In densification, there is increased matrix viscosity, reduced interfascial glide, impaired force transmission, and consequent irritation of free nerve endings. These changes can be visualized in vivo using high-resolution ultrasound and sonoelastography [76]. Sonoelastography studies reveal that stiffness distribution in fascia and muscle is heterogenous in both hypermobile and non-hypermobile populations. In hEDS, both fascia and muscle often demonstrate abnormal softening, whereas in non-hEDS individuals with musculoskeletal pain, fascia is softened while the associated muscle (e.g., perimysium and epimysium) is often stiffer [74].

Changes in ECM composition and viscosity are thought to impair shear strain—interfascial gliding [77], which in turn disrupts movement mechanics and may contribute to symptoms of impaired proprioception, altered biomechanics, and chronic pain. Specifically, prior studies have shown reduced interfascial glide in the tensor fascia lata of hEDS/HSD individuals, consistent with findings of excessive myofibroblast density in the iliotibial tract [27,73].

This densification and impaired fascial glide can irritate free nerve endings, affect gamma-motor regulation, and disrupt mechanosensory integration, potentially contributing to the impaired proprioception, altered gait mechanics, and chronic pain commonly observed in hEDS and HSD patients [78,79,80]. Abnormal shear strain, reduced interfascial movement, and increased ECM volume have all been correlated with increased pain [74,81,82]. In addition, abundant C-fibers embedded in the fascia are sensitive to ECM changes, and excessive matrix deposition may directly irritate these fibers, contributing to widespread pain [57].

The complex of muscles, tendons, and connective tissues surrounding a joint contribute to joint stabilization [83]. Densification in the peri-joint structures can alter biomechanics and exacerbate joint incoordination and pain. Specifically, densification in the pelvic region can affect lumbopelvic stability [84,85]. The lateral third of the fascia iliacus contributes to the conjoint tendinous sheet shared with the transversus abdominis and internal oblique muscles, which play a crucial role in lumbar spine stability [86,87,88]. Therefore, changes in fascial structure in the hip and pelvis region may disrupt fascial continuity, force transmission, and muscular coordination, compromising core stability and contributing to lumbar spine dysfunction, a common site of impairment in this population [89]. Collectively, these findings highlight the role of fascial and myofascial integrity in the maintenance of joint and spine coordination, and their relevance in the symptomatic manifestations of hEDS and HSD.

These structural changes with associated core instability likely contribute to biomechanical deviations such as Trendelenburg gait, characterized by pelvic drop and compensatory weight shifting, which increases tension on the iliotibial and lumbopelvic fascia [90,91,92]. The pathologic movement patterns likely lead to further stress on myofibroblasts. Mechanical stress is a key driver of fascial densification. Fascia is a mechanically responsive tissue, and its cells—specifically fibroblasts—are highly sensitive to physical tension. This mechanosensitivity allows for fibroblasts to adapt to the needs of different tissues in response to the different loads they receive [93]. Immunohistochemical studies have demonstrated that myofibroblasts are more prevalent in areas subjected to mechanical tension with strong crimp formation of the collagen fibers, such as lumbar fascia and intramuscular perimysium [94,95]. Localized repetitive strain overstimulates mechanosensitive fibroblasts, triggering excessive reinforcement of the ECM in specific areas [92,96]. The presence of myofibroblast in the deep fascia of this population suggests that these mechanical stressors are plausible contributors to the pathological process [27].

In response to mechanical overload, the fascia may undergo compensatory remodeling known as stress shielding. In this process, thickened fascia assumes a greater share of load-bearing responsibility, reducing the activation and loading of adjacent stabilizing muscles. Over time, this leads to muscle underuse, weakness, and eventual sarcopenia [97,98]. In hEDS/HSD, where hypotonia and joint instability are already present, stress shielding can further destabilize the musculoskeletal system, creating a feedback loop of dysfunction.

Altered biomechanical strain and compromised fascial mechanics may contribute to a higher rate of joint instability [75]. These muscle–fascia interactions can also affect joint mechanics through impaired force transmission through myofascial expansions—fascial extensions that distribute mechanical force across adjacent tissues. These expansions contribute to over 30% of mechanical force transmission and are critical for coordinated movement [99,100,101]. In hEDS/HSD, impaired fascial stiffness and gliding may interrupt this force transmission, concentrating stress at entheses sites and contributing to enthesopathies and joint instability [78,79,102].

In sum, the structural changes observed in the deep fascia of individuals with hEDS and HSD reflect a maladaptive response to inflammation, mechanical stress, and immune dysregulation. These changes compromise biomechanical efficiency, interfere with force transmission and proprioceptive feedback, and perpetuate chronic pain and systemic dysfunction.

### 3.2. Superficial Fascia Involvement

Emerging evidence suggests that co-occurring adipose disorders such as lipedema and Dercum disease are associated with distinct patterns of fascial remodeling in individuals with hEDS. Alterations in both superficial and deep fascia may provide critical insight into the pathophysiology and clinical presentation of these complex, overlapping conditions.

Individuals with hEDS and co-occurring adipose disorders exhibit a significantly increased thickness of both deep and superficial fascia compared to those with hEDS alone. Ultrasound imaging reveals that there is correlated superficial fascial thickening and deep fascial thickening, suggesting a continuum of connective tissue remodeling that spans multiple fascial layers [103]. These findings support the hypothesis that adipose disorders such as lipedema and Dercum disease involve fascial remodeling that extends beyond adipose hypertrophy.

Previous studies in this population with co-occurring hypermobility disorder and adipose disorder show a correlation between immune dysfunction and pre-tibial superficial fascia thickness [103]. This suggests that immune dysregulation contributes to superficial fascial changes in individuals with co-occurring hEDS and adipose disorders. Both lipedema and Dercum disease are associated with chronic, low-grade inflammation mediated by M2 macrophages. These cells promote angiogenesis, interstitial fluid accumulation, and ECM remodeling. The presence of M2 macrophage-mediated inflammation supports the view that these conditions are not isolated adipose disorders but rather systemic fascial pathologies [104]. The resulting accumulation of proteoglycans and profibrotic ECM components likely contributes to both deep and superficial fascial thickening.

Furthermore, a moderate correlation was also observed between immune dysfunction and dysautonomia in hEDS with co-occurring adipose disorder, consistent with previous studies of hEDS populations without adipose disorders [56,103]. In both hEDS and adipose disorders, there is a large burden of autonomic dysfunction, with symptoms including shortness of breath, abdominal pain, cognitive dysfunction, migraines, easy bruising, and varicose veins. These correlations underscore the complex interplay between immune function, connective tissue integrity, and autonomic regulation in hypermobility-related syndromes.

### 3.3. Structural and Functional Changes in Tendons

One of the features of hEDS is joint laxity, particularly affecting tendons and ligaments. Tendons serve as dynamic stabilizers of joints, yet limited studies have examined their structural and functional characteristics in hEDS and HSD, with often inconsistent findings [74,105,106].

Reduced tendon stiffness in hEDS has been linked to increased tendon elongation (10.1% to 21.8%), patellar hypermobility, and risk of subluxation or dislocation [107,108,109]. Dynamic assessments have shown that individuals with hEDS exhibit a 27% lower secant modulus at common force and 34% lower at maximal force compared to controls, reflecting significantly reduced tendon stiffness and increased deformation under load [110].

This can compromise joint stability, functional strength, and neuromuscular coordination [111,112,113]. Tendon stiffness modulates force transmission in the muscle–tendon unit. While tendons do not generate force, they influence the muscle’s ability to produce and transmit force efficiently [114,115]. Imbalances between muscle force production and tendon compliance disrupt the muscle–tendon unit, affecting the storage and release of elastic energy [116,117]. This may underlie common gait abnormalities observed in hEDS, including reduced walking speed, stride length, and step length.

Although the etiology of tendon pathology is multifactorial [118,119], in hEDS, this strain-induced tissue damage may be a significant risk factor for tendinopathy development [120,121,122]. Tendons adapt to load via Piezo1-mediated mechanotransduction [93,123,124]. However, tendon adaptation occurs more slowly than in muscle [125,126,127], and in hEDS, the native high elasticity of tendons further complicates this balance. Optimal strain for tendon adaptation typically lies between 4.5% and 6.5% using 90% of isometric maximum voluntary contraction (MVC) [128,129]. When muscle force is generated without sufficient corresponding tendon stiffness, tendons are subjected to high strain levels during muscle contraction [130]. Repetitive strains above 9.0% degrade tendon structure and impair its micromorphology, compromising tendon integrity [122,131,132]. In hEDS, tendon elongation is between 10.1% to 21.8% at MVC [107]. These values represent excessive strain, accelerated tissue failure, and reduced time to rupture during both static and cyclic loading [133], leading to frank dislocations. On the other hand, insufficient strain (<3.0%) (like lack of loading in those with dysautonomia or post-surgery) can also activate catabolic pathways, further deteriorating tendon quality [122].

Enthesopathy is a common sonographic finding in hEDS and reflects underlying ECM dysfunction [75]. Enthesopathy is characterized by chronic degenerative changes including collagen disorganization, increased water content, and inflammation [45]. These changes are frequently linked to repetitive microtrauma and impaired wound healing due to connective tissue fragility and joint hypermobility [134,135,136]. Increased patellar tendon thickness and elevated hypoechogenicity observed in hEDS and HSD populations likely result from these intrinsic biomechanical and molecular alterations, predisposing individuals to tendinopathy and impaired function [75,137].

Resistance training is one of the few interventions shown to increase tendon stiffness in hEDS [107,138]. In contrast, walking—although widely prescribed in this population and necessary for cardiovascular training—produces peak ground reaction forces (1.2–2× body weight) that are likely insufficient to stimulate tendon adaptation [139,140]. Targeted resistance regimens using high-load, low-repetition protocols are more effective at enhancing tendon properties than low-load, high-repetition exercises [121,141,142,143]. However, care must be taken not to overload tendons in this population; tendon elongation above optimal strain thresholds compromises micromorphology and integrity, making proper load dosing essential [122,131,132,144].

## 4. Fascial Changes in hEDS/HSD Compared to Mimicking Conditions

Fascial alterations in hEDS/HSD exhibit unique pathophysiological characteristics that can help distinguish these conditions from clinically overlapping or mimicking disorders, such as spondyloarthritis, fibromyalgia, and other hereditary connective tissue diseases like Marfan syndrome.

### 4.1. Other Hereditary Connective Tissue Disorders

While both hEDS and Marfan syndrome are heritable connective tissue disorders, their underlying mechanisms and clinical manifestations diverge significantly. In both conditions, studies have identified compromised biomechanical properties such as reduced elastic modulus in the patellar tendon [110]. However, Marfan syndrome is specifically associated with mutations in the fibrillin-1 gene, which lead to microfibrillar structural defects, such as thinner fibers and irregular “beads-on-a-string” morphology and dimensions. These abnormalities contribute to a weakened extracellular matrix scaffold [145].

Fibrillin-1 is expressed in the epimysium and perimysium, and its deficiency disrupts the organization and elasticity of the connective tissue matrix. Muscle biopsies in individuals with Marfan syndrome confirm reduced fibrillin immunoreactivity, myopathic changes, and increased fibrosis. Histological studies demonstrate increased collagen deposition and fibrosis within these fascial layers, resulting in fibrotic niches that impair satellite cell proliferation and differentiation, leading to muscle atrophy and weakness [146,147].

Clinically, this manifests as reduced muscle mass, diminished strength, and progressive functional impairment. These features contrast with hEDS, where the primary pathology is due to extracellular matrix disorganization, tissue fragility, and densification, rather than fibrillin-mediated fibrotic degeneration.

### 4.2. Autoimmune Conditions

In spondyloarthritis and other autoimmune conditions, the enthesis is a key site of immune activation. Enthesopathy in spondyloarthritis is typically characterized by calcifications, enthesophyte formation, and localized edema, resulting from a combination of mechanical stress and immune-mediated inflammation [148]. These inflammatory changes contrast with those observed in hEDS, where enthesopathy primarily arises from tissue fragility, ligamentous laxity, and joint instability, with minimal levels of calcific changes [75].

Previous studies have demonstrated that thoracolumbar myofascial elasticity in spondyloarthritis negatively correlates with disease duration, suggesting progressive myofascial stiffness associated with chronic inflammation [149]. In contrast, individuals with hEDS tend to exhibit greater fascial elasticity or reduced stiffness independent of disease duration. This pattern likely reflects a complex interplay of biomechanical instability, connective tissue fragility, and neurogenic factors, rather than a primarily inflammatory process [74].

In cases where spondyloarthritis co-occurs with hEDS, compounded fascial densification may occur, likely representing the additive effects of inflammation and biomechanical stresses. Moreover, while biologic therapies are effective in reducing systemic inflammation and symptomatic disease activity in autoimmune conditions, current evidence suggests that they do not reverse fascial remodeling, indicating that structural changes within the fascia may persist despite immunosuppressive therapy [75].

In systemic lupus erythematosus, skin biopsies typically show dermatitis with a vacuolar alteration of basal keratinocytes, perivascular and periadnexal lymphocytic infiltrates, basement membrane thickening, and dermal mucin deposition. Direct immunofluorescence often reveals granular IgG, IgM, and C3 deposition at the dermal–epidermal junction [150]. In rheumatoid arthritis, rheumatoid nodules are characterized histologically by central fibrinoid necrosis surrounded by palisading histiocytes and chronic inflammatory cells. Other findings may include granulomatous inflammation, neutrophilic dermatoses, vasculitis, and vascular thrombosis, typically without dermal–epidermal junction involvement [151]. These immunological changes are not present in HSD/hEDS.

In idiopathic inflammatory myopathies, such as dermatomyositis and antisynthetase syndrome, immune-mediated injury targets the perimysium and perifascicular regions. Muscle biopsy frequently reveals perimysial inflammation, connective tissue fragmentation, and perifascicular atrophy [152]. On imaging, this pathology presents as a “honeycomb” pattern of myofascial edema—reticular or patchy increased signal intensity on T2-weighted or STIR sequences [153]. In contrast, hEDS/HSD does not typically show immune cell infiltration or inflammatory fascial edema on biopsy or imaging.

### 4.3. Fibromylagia

Fibromyalgia shares several symptomatic overlaps with hEDS/HSD, including widespread pain, tenderness, and fatigue. However, the underlying mechanisms appear distinct. Biopsies from fibromyalgia patients demonstrate increased oxidative stress and elevated inflammatory markers in the dorsal root ganglia, implicating both central sensitization and peripheral neuroinflammation [154].

Further supporting this pathophysiology, serum IgG from fibromyalgia patients can induce pain hypersensitivity when transferred to mice [155]. This finding strongly suggests an autoimmune or neuroimmune component to fibromyalgia, differentiating it from the biomechanical and extracellular matrix-driven alterations observed in hEDS/HSD. Fascial involvement in fibromyalgia may be secondary to neurogenic inflammation and dysregulated pain processing, rather than due to structural extracellular matrix abnormalities or connective tissue fragility.

## 5. Implications of Disease Progression, Aging, and the Fascial System in hEDS/HSD

Although symptoms often begin in childhood or adolescence, hEDS/HSD diagnosis is often delayed until adulthood due to the subtlety and variability of early clinical signs [156]. This is complicated by changes in joint hypermobility with age. In the general population, joint hypermobility typically declines with age, as reflected by decreasing Beighton scores and a histological reduction in elastic fibers within the deep fascia [157,158]. While the number of hypermobile joints may decline with age, in hEDS, generalized joint laxity persists into adulthood. In individuals with hEDS/HSD, this expected decline paradoxically coincides with the onset or worsening of joint instability, reduction in fascial glide, and chronic pain syndromes.

In early life, generalized joint laxity in hEDS/HSD is often associated with recurrent microtrauma, aberrant mechanotransduction, and persistent low-grade immune activation [79]. These processes contribute to long-term dysregulation of the ECM. This is further compounded by chronic maladaptive loading, leading to microinjury, progressive ECM remodeling, and fascial dysfunction [41]. The resulting tissue dysfunction impairs proprioception and motor coordination, activates nociceptive pathways, and disrupts the organization of deeper fascial layers.

Histologically, concurrent aberrant integrin signaling and activation of the Wnt/β-catenin pathway exacerbate ECM dysregulation. Wnt pathway activation increases the expression of matrix-degrading enzymes and disrupts ECM architecture, contributing to the tissue fragility characteristic of hEDS and HSD [30].

Clinically, the interaction between persistent joint laxity and tissue fragility with ongoing fascial remodeling leads to evolving phenotypes over time. Importantly, reductions in joint range of motion with age do not necessarily reflect the resolution of hypermobility disorder but may instead indicate pathological changes in fascial composition and mechanics. In some individuals, particularly those with a historical HSD, this may manifest as a transition from soft tissue laxity to progressive fascial stiffening with loss of joint hypermobility. Others may retain pronounced tissue laxity and architectural disorganization throughout life [37,79].

Concurrently, fluctuating tissue and joint laxity may occur in response to comorbidities or chronic inflammatory states. Individuals with poorly controlled comorbidities or systemic inflammation may exhibit exaggerated tissue laxity. Oscillations between stiffness and laxity can be influenced by trauma, immune dysregulation, hormonal fluctuations, and environmental factors [103]. Some individuals respond favorably to load-based rehabilitation that increases fascial stiffness and manual therapy that enhances fascial glide, leading to functional improvement and partial normalization of joint mobility [82,107]. These factors collectively shape fascial integrity and joint mobility, highlighting the heterogeneity and complexity of disease progression in hEDS and HSD.

Ultimately, the progression from childhood laxity to adult fascial dysfunction in hEDS and HSD reflects a multifactorial process involving biomechanical stress, immune activation, fibroblast behavior, and chronic ECM remodeling. These complex dynamics underscore the need for age- and stage-specific assessment of hypermobility, as well as longitudinal monitoring and individualized management strategies.

## 6. Conclusions and Future Directions

This review underscores the growing recognition of hEDS and HSD as systemic connective tissue disorders rooted in fascia-centered pathophysiology. The convergence of fibroblast-to-myofibroblast transition, dysregulated ECM remodeling, immune dysfunction, and altered mechanotransduction pathways presents a unifying histological and anatomical framework for understanding the complex clinical presentations observed in these conditions. 

A key insight emerging from the literature is that fascia is not a passive structural element but an active tissue involved in mechanosensation, immune modulation, force transmission, and fluid balance. Aberrant fibroblast behavior, sustained myofibroblast activation, and increased ECM turnover contribute to widespread tissue fragility, chronic pain, proprioceptive dysfunction, and autonomic symptoms [7,18,19]. Moreover, the co-occurrence of adipose disorders such as lipedema and Dercum disease highlights the need for integrated diagnostic frameworks that account for overlapping fascial, vascular, and inflammatory mechanisms [103].

Despite these advances, several critical gaps remain. Histological studies in hEDS and HSD are still limited, particularly with regard to the superficial and deep fascia, entheses, and tendon-to-muscle junctions and their direct implication in joint stability [107,108,109]. Longitudinal research examining the progression of fascial and tendon pathology over time—and in response to therapeutic interventions—is urgently needed.

One of the most significant opportunities for future research lies in understanding the histologic impact of targeted exercise interventions. While resistance training has shown promise in improving tendon stiffness and function, the cellular and molecular adaptations to mechanical loading in hEDS/HSD remain largely unexplored [107,138]. Specifically, studies are needed to determine how varying load intensities, time-under-tension protocols, and recovery durations influence fibroblast activity, myofibroblast persistence, ECM turnover, and collagen microarchitecture in affected tissues. Advanced imaging modalities (e.g., sonoelastography, MRI elastography) combined with tissue biopsies could provide insight into the structural and molecular changes induced by exercise [18,27].

Additionally, the role of pharmacologic interventions in modulating fascial remodeling is a promising but under-investigated area. Agents that influence TGF-β signaling, mast cell stabilization, Wnt/β-catenin pathways, or MMP activity could theoretically attenuate pathological tissue remodeling, yet no clinical trials have systematically evaluated such therapies in hEDS or HSD [45,46]. Emerging biologics and small molecules currently under study in fibrotic and inflammatory conditions may offer translational potential in the context of hypermobility syndromes [3].

Furthermore, studies investigating sex-specific responses, hormonal influences, and the effects of comorbid dysautonomia and immune dysfunction on tissue repair and exercise tolerance will be essential for developing personalized treatment strategies. Optimizing the dose–response relationship of both pharmacologic and mechanical stimuli is critical for restoring function while minimizing risk.

hEDS and HSD represent complex multisystem disorders characterized by profound alterations in fascial structure, function, and regulation. While current insights provide a compelling fascia-centered model, translational studies integrating histological, biomechanical, and clinical data are urgently needed. A multidisciplinary research agenda—linking molecular biology, exercise physiology, rehabilitation science, and connective tissue pathology—will be essential to advancing care for individuals living with these underrecognized but deeply impactful disorders.

## Figures and Tables

**Table 1:** Summary of key pathophysiological manifestations in hEDS and HSD.

Level	Dysfunction	Mechanism
Molecular Level	Molecular Pathways	Key molecular dysregulation includes aberrant activation of Wnt/β-catenin signaling, altered miRNA expression (e.g., downregulation of miR-23a, upregulation of miR-224 and miR-378-3p), upregulation of prolactin receptor (PRLR), and decreased NR4A nuclear receptor expression, contributing to inflammation, myofibroblast persistence, and ECM disorganization.
Cellular Level	Fibroblast Dysfunction	Pathological fibroblast-to-myofibroblast transition is driven by the αvβ3 integrin–ILK–Snail1/Slug signaling axis, resulting in persistent α-SMA expression, resistance to apoptosis, and enhanced migratory and matrix-altering capacity.
	ECM Remodeling	Collagen I/III/V are aberrantly organized; elastin is mislocalized within cells; fibronectin fails to integrate and fragments. There is also downregulation of integrins α2β1 and α5β1 and fibronectin receptor dysfunction, disrupting ECM–cell communication.
	Immune Dysregulation	Persistent immune–fibroblast crosstalk, involving mast cells, macrophages, and T-cells, leads to elevated levels of IL-6, TGF-β1, MCP-1, and ECM-derived DAMPs, reinforcing chronic inflammation, tissue fragility, and immune hypersensitivity.
Tissue Level	Fascial Layer Alterations	Sonoelastography reveals fascial thickening, reduced interfascial glide, and altered viscoelasticity. Increased myofibroblast density is seen in high-load fascial regions, contributing to densification, pain, and reduced proprioception.
	Tendon and Enthesis Involvement	Tendons exhibit reduced stiffness and excessive elongation (10.1–21.8%) impairing force transmission and increasing the risk of subluxation and dislocation. Enthesopathies are common due to collagen disarray, water retention, and repetitive microtrauma
	Adipose Disorders	Lipedema and Dercum disease frequently co-occur and are associated with thickened superficial fascia, M2 macrophage-dominant inflammation, proteoglycan accumulation, and increased autonomic and immune dysregulation.
System Level	Dysautonomia	Approximately 60% of individuals with hEDS/HSD experience autonomic symptoms, likely due to dense autonomic innervation of fascia and TGF-β1-mediated feedback loops triggered by emotional stress, tissue strain, and immunological changes.
	Immune Dysregulation	Mechanical strain/injury, psychological stress, autonomic imbalance, and immune activation converge through shared signaling pathways (e.g., TGF-β1 and prolactin), reinforcing fibroblast activation and systemic inflammation.
Clinical Expression	Aging and Disease Progression	Over time, symptoms often shift from hypermobility to changes in gliding property and joint instability. These phenotypic changes reflect accumulated microtrauma, altered ECM remodeling, inflammation, and mechanical compensations, especially in fascia and tendons. Hypermobility and clinical expression may vary with immune state, trauma, and hormones.

## Abbreviations

The following abbreviations are used in this manuscript:

hEDS	Hypermobile Ehlers–Danlos Syndrome
EDS	Ehlers–Danlos Syndrome
HSD	Hypermobility spectrum disorder
ECM	Extracellular matrix
